# Quality of child and adolescent care transitions considering the presence of chronic disease

**DOI:** 10.1590/0034-7167-2022-0347

**Published:** 2023-05-29

**Authors:** Vitória Carolini Gomes, Gabriela Marcellino de Melo Lanzoni, Caroline Cechinel-Peiter, José Luís Guedes dos Santos, Ana Lúcia Schaefer Ferreira de Mello, Aline Lima Pestana Magalhães

**Affiliations:** I Universidade Federal de Santa Catarina. Florianópolis, Santa Catarina, Brazil

**Keywords:** Nursing, Chronic Disease, Continuity of Patient Care, Child Health, Transitional Care, Enfermería, Enfermedad Crónica, Continuidad de la Atención al Paciente, Salud del Niño, Cuidado de Transición, Enfermagem, Doença Crônica, Continuidade da Assistência ao Paciente, Saúde da Criança, Cuidado Transicional

## Abstract

**Objectives:**

to analyze the quality of child and adolescent care transitions from hospital to home, considering the presence of chronic disease.

**Methods:**

quantitative, cross-sectional study, carried out from February to September 2019 in two hospitals in the south of Brazil. We used an instrument to characterize participants and the Care Transitions Measure (CTM-15) for the legal tutors of children and adolescents that were discharged from the institutions.

**Results:**

the general mean of the quality of transition of care was 87.9 (SD=13.4), in a scale from 0 to100). We found a significant difference in the quality of transition of care when comparing patients with and without chronic disease (90.0 and 84.3; p=0.001).

**Conclusions:**

we found the quality of the transition of care to be satisfactory, with better results for patients with chronic disease. This can help understand the most impactful aspects in the transition of care, especially in regard to children health.

## RESUMO

**Objetivos:**

analisar a qualidade da transição do cuidado de crianças e adolescentes do hospital para o domicílio considerando a presença de doença crônica.

**Métodos:**

estudo quantitativo, transversal, realizado de fevereiro a setembro de 2019 em dois hospitais no Sul do Brasil. Foram aplicados instrumento de caracterização e o *Care Transitions Measure* (CTM-15) a responsáveis legais de crianças e adolescentes que tiveram alta das instituições.

**Resultados:**

a média geral da qualidade da transição do cuidado foi de 87,9 (DP=13,4) em escala de 0 a 100 pontos. Foi verificada diferença na qualidade da transição do cuidado entre pacientes com e sem doença crônica (90,0 e 84,3; p=0,001).

**Conclusões:**

a qualidade da transição do cuidado foi considerada satisfatória, sendo mais bem avaliada nos casos de pacientes com doença crônica, o que pode orientar a compreensão de aspectos influentes na transição do cuidado, sobretudo no tocante à saúde da criança.

## RESUMEN

**Objetivos:**

analizar calidad de la transición del cuidado de niños y adolescentes desde el hospital al domicilio considerando la presencia de enfermedad crónica.

**Métodos:**

estudio cuantitativo, transversal, realizado de febrero a septiembre de 2019 en dos hospitales del Sur brasileño. Aplicados instrumento de caracterización y *Care Transitions Measure* (CTM-15) a responsables legales de niños y adolescentes que tuvieron alta de las instituciones.

**Resultados:**

la mediana general de la calidad de la transición del cuidado fue de 87,9 (DP=13,4) en escala de 0 a 100 puntos. Verificada diferencia en la calidad de la transición del cuidado entre pacientes con y sin enfermedad crónica (90,0 y 84,3; p=0,001).

**Conclusiones:**

la calidad de la transición del cuidado fue considerada satisfactoria, siendo mejor evaluada en casos de pacientes con enfermedad crónica, lo que puede dirigir la comprensión de aspectos influyentes en la transición del cuidado, sobretodo en lo que respecta a la salud infantil.

## INTRODUCTION

When the transitions between services is frequent, the fragmentation of care becomes more likely, causing issues in patient health management, caregiver work overload, and increasing the frequency of rehospitalizations and the cost of health^(1)^. After hospital discharge, home care demands time and dedication from those involved, as it demands constant monitoring of the patient due to its complexity and duration. Thus, it overloads caregivers and leads to a diminution in the general wellbeing of the patient, especially of those who are experiencing some chronic disease^(2)^. To overcome these events, we must take advantage of some strategies, such as the transition of care^(1)^.

**Chart 1:** Identification of the items in the instrument Care Transitions Measure (CTM-15), Florianópolis, Santa Catarina, Brazil, 2022

Items		Fator
1.	Agreed with the health care team on health goals and means	3
2.	Preferences considered when deciding health needs	3
3.	Preferences considered when deciding where health needs are met.	3
4.	Had information needed for self-care	1
5.	Understand how to manage health	1
6.	Understand warning signs and symptoms	1
7.	Had a written care plan	4
8.	Understand what makes the health condition better or worse	1
9.	Understand health care responsibilities	1
10.	Confident knew what to do to manage care	1
11.	Confident could do what needed to take care of health	1
12.	Had a written list of appointments or exams for the next weeks	4
13.	Understand medications’ purpose	2
14.	Understand how to take medications	2
15.	Understand medications’ side effects	2

This transition may involve several strategies, such as planning the discharge, early planning of care, educational actions for the safe use of medication, complete communication of information, outpatient or telephone follow up with patients, health education, preparing for self-management, use of operational protocols, structuring support networks, reviewing operational procedures, and organizing referral and counter-referral systems. These models must be standardized in the health institution to guarantee the quality of care transition^(3-4)^.

A retrospective study carried out in Brazil found that 17.1% of children attended in a pediatric hospitalization unit for a period of two years had to be hospitalized more than once in the period^(5)^. It should be mentioned that some rehospitalizations can be avoided through effective actions of transition of care, which guarantee the behavior needed for the patient in the several health services, reducing avoidable risks associated with hospitalization and unnecessary costs to the health systems^(3)^. As a result, the implementation of care transition services reduces the rates of rehospitalization in health care units^(6)^.

In this context, the importance of integrating the Health Care Network (HCN) stands out, since it is a focus point in the Primary Health Care (PHC), where it coordinates care and uses other care units as support^(7)^ to promote the continuity of care in health services with different technologies available to them.

In health services, the follow up of patients with chronic diseases requires special care so continued support can be offered. The constant possibility of events requires continuous observation and attention from many different professionals in several health care contexts^(1)^. Chronic disease in children, especially, have a direct impact on the quality of life of children and their families, with potential physical, mental, cultural, and social repercussions^(2)^.

Still, when the HCN is not operationalized as prescribed, the PHC often cannot attend to all the demand of chronic disease patients. Providing integral care to children with these diseases depends on the articulation the Health Care Network, to allow for adequate communication flow and access to the many points of the network the patient needs, including the transition of care after discharge. Otherwise, it would be impossible for the patient to experience the continuity of care^(8)^.

In literature, the transition of the care of pediatric patients is still seldom explored. Few studies that evaluate the quality of this transition were found in international literature^(9-10)^, none in Brazil.

Considering the context above, our research question is: How does the transition of care of children and adolescents from hospital to home take place, considering the presence of chronic disease?

## OBJECTIVES

To analyze the quality of child and adolescent care transitions from hospital to home, considering the presence of chronic disease.

## METHODS

### Ethical aspects

This research is part of a larger project named “Continuity and Transition Care in the Health Care Network”, which was approved by a Research Ethics Committee for Research with Human Beings, according with Resolution 466/2012 from the National Council of Health. All participants signed the Free and Informed Consent Form.

### Design, period, and place of study

This is a quantitative, analytical, and cross-sectional research. Data collection took place from February to September 2019 in two large hospitals in the south of Brazil, including one pediatric hospital and the pediatric hospitalization unit of a general hospital. Both institutions are references in the state for high complexity care. The recommendations from the STROBE were followed in the methodology of this study.

### Population; criteria of inclusion and exclusion

Inclusion criteria were: tutors of patients aged below 18 years hospitalized for 24 hours or more in the institutions included in the investigation. We excluded the patients who had died during hospitalization, had been transferred to other units, or readmitted after discharge; children younger than 29 days; and those whose tutors did not answer telephone contact.

The minimum sample size calculation considered the hospitalization rates of the hospitals, with a confidence level of 95%, margin of error of 4 points, mean of 74.7, and standard deviation of 17.1^(11)^. The calculations resulted in a minimum sample of 156 participants.

From the 395 participants invited to participate in the study, 73 did not answer the telephone, 9 were tutors of children aged less than 29 years, 3 had been transferred into other hospitals, 2 had died, 2 abandoned the research, and 1 was readmitted after discharge. This led to a total of 305 participants.

### Study protocol

We applied an instrument to characterize the participants and a version of the Care Transitions Measure (CTM-15) adapted to the Brazilian context. Data collection was divided in two stages. The first included addressing the patients and tutors in the hospital during hospitalization, when they were invited to participate in the research. After they agreed, we applied instrument to characterize the participants, which included the following variables: gender, city of residence, connection with the patient, whether the patient had a chronic disease according to a ICD-10 disease. To determine the chronic diseases, the most commonly present in children hospitalized in Brazil were considered^(12)^.

The second stage of data collection took place from 7 to 30 days after discharge, through telephone. To evaluate the quality of the care transition from hospital to home, from the perspective of the patient or caregiver, the CTM-15 was used. This instrument was developed in 2002, in the United States, and, in 2016, translated and validated for use in Brazil. It includes 1 5 items whose answers are in a 4-point Likert scale.

Each item has five possible response options: Strongly agree; Disagree; Agree; Strongly Agree; and I do not know/do not remember/does not apply. The last option has no effect on the final calculation of the mean. The CTM-15 items are divided in four factors: 1) Management preparation; 2) Understanding medications; 3) Preferences imported; and 4) Care plan.

The final score is transformed in a linear scale using the formula [(*score* - 1)/3] * 100, where the higher the linear scale means, the higher the care transition quality^(13)^.

### Analysis of results and statistics

Data was organized and processed using the software Statistical Package for the Social Sciences (SPSS), where we applied Student’s t test and the analysis of variance (ANOVA). We considered a level of significance of 5% (p<0.05).

## RESULTS

Regarding the participants of the study, most were male (172; 56.4%), lived in the same city of the hospitals (133; 43.6%) and had some form of chronic disease (192; 63.0%). In most cases, the mother was the tutor present (233; 76.4%). The most common chronic maladies were respiratory system diseases (62; 32.3%, followed by congenital malformations, deformations, and chromosomal abnormalities (58; 30.2%) (Table 1).

**Table 1:** Characterization of the participants included in the research, Florianópolis, Santa Catarina, Brazil, 2019

Variable	Category	n	%
Gender	Male	172	56.4
	Female	133	43.6
City of residence	Same city of the hospitals	133	43.6
	Same health region of the hospitals	101	33.1
	Other cities	71	23.3
Connection between the tutor and the child	Mother	233	76.4
	Father	44	14.4
	Others	28	9.2
Chronic disease	Yes	192	63.0
	No	113	37.0
Type of chronic disease according with ICD 10 chapter	Respiratory system diseases	62	32.3
	Congenital malformations, deformations, and chromosomal abnormalities	58	30.2
	Neoplasms	19	9.9
	Diseases of the circulatory system	11	5.7
	Diseases of the nervous system	11	5.7
	Endocrine, nutritional, and metabolic diseases	9	4.7
	Disease of the digestive system	8	4.2
	Diseases of the genitourinary system	5	2.6
	Mental and behavioral disorders	7	3.7
	Disease of blood and blood-forming organs, and some immune disorders	2	1.0

The total mean of the CTM-15 was 87.9. The total mean among patients with chronic diseases was 90.0, versus 84.3 among those with no disease. The item 9 of the CTM-15 showed the highest mean among patients, 94.7, followed by the item 14, whose mean was 94.3. The lowest mean was in item 15, with 67.8. In items 3, 12, 14, and 15, there was a difference in the mean result of patients with chronic disease and patients those who did not have a chronic disease (Table 2).

**Table 2:** Mean of care transition quality according with CTM-15 items, Florianópolis, Santa Catarina, Brazil, 2019

CTM-15 items	Total		Patients with chronic disease		Patients with no chronic disease		*p* value
	Mean	Standard deviation	Mean	Standard deviation	Mean	Standard deviation	
1	87.6	18.9	89.2	17.7	85.0	20.4	0.065
2	86.4	24.4	88.4	22.9	83.0	26.5	0.078
3	84.1	25.0	86.9	23.0	79.5	27.7	0.018
4	90.3	20.0	91.6	18.8	88.2	21.8	0.171
5	92.1	18.1	93.2	17.3	90.3	19.3	0.191
6	91.5	18.9	92.8	17.8	89.4	20.5	0.142
7	82.9	28.6	84.1	29.0	80.9	28.0	0.349
8	91.8	17.2	93.3	15.0	89.3	20.1	0.066
9	94.7	12.5	95.6	11.3	93.2	14.2	0.120
10	89.4	20.1	90.9	18.7	86.9	22.1	0.112
11	90.1	18.3	90.9	17.4	88.7	19.8	0.318
12	82.0	29.1	87.3	25.9	72.8	31.9	<**0.001**
13	91.2	19.5	92.7	19.6	88.7	19.1	0.095
14	94.3	16.0	96.0	13.0	91.3	19.9	0.038
15	67.8	38.0	76.1	34.9	52.9	39.0	<**0.001**
Total	87.9	13.4	90.0	11.9	84.3	15.1	**0.001**

* *Student's t test.*

Regarding CTM-15 factors, Factor 1 Management Preparation, had the highest mean, with 91.4, while the lowest mean was 82.6, in Factor 4, Care Plan. Factors 2 (Understanding Medications), 3 (Preferences Imported), and 4 (Care Plan) had a significant difference between the groups, indicating that the care transition of patients with chronic disease was of a higher quality (Table 3).

**Table 3:** Mean of care transition quality according with CTM-15 factors, Florianópolis, Santa Catarina, Brazil, 2019

CTM-15 factors	Total		Patients with chronic disease		Patients with no chronic disease		*p* value
	Mean	Standard deviation	Mean	Standard deviation	Mean	Standard deviation	
1	91.4	13.7	92.6	12.1	89.3	16.0	0.058
2	84.8	18.5	88.5	17.6	78.5	18.4	<**0.001**
3	86.0	18.2	88.1	16.5	82.5	20.4	**0.014**
4	82.6	23.4	85.5	22.6	77.5	24.0	**0.004**

* *Student's t test.*

Regarding the type of chronic disease the child presented, the CTM-15 means varied from 94.9 among those with circulatory system diseases to 80.0 in those with disease of blood and blood-forming organs, and some immune disorders. Nonetheless, this difference was not significant (Table 4).

**Table 4:** Mean of care transition quality considering the ICD-10 chapter about the chronic disease, Florianópolis, Santa Catarina, Brazil, 2019

Type of chronic disease according with ICD10 chapter	Total mean	Standard deviation	*p* value
Respiratory system diseases	88.8	12.5	0.480
Congenital malformations, deformations, and chromosomal abnormalities	90.6	11.1	
Neoplasms	92.5	7.6	
Diseases of the circulatory system	94.9	4.3	
Diseases of the nervous system	91.1	12.0	
Endocrine, nutritional, and metabolic diseases	92.4	12.2	
Disease of the digestive system	90.5	10.6	
Diseases of the genitourinary system	87.6	16.5	
Mental and behavioral disorders	82.3	21.1	
Disease of blood and blood-forming organs, and some immune disorders	80.0	28.3	

* *ANOVA.*

## DISCUSSION

Most patients included in the study had some chronic disease. This prevalence may be associated with the characteristics of the patients who were referred to the hospitals where the study took place: the pediatric hospitalization unit of a general hospital and that of a children’s hospital, both state references for several specialties, which directs the level of complexity of the service provided by each hospital^(5)^.

Most participants were accompanied by their mothers. Strictly speaking, they are more involved with the care of the child than the other members of the family^(14)^. For mothers, their role as main caregiver means they have to carry out activities in addition to those of their daily lives, which causes stress and hinders other aspects of their lives - such as their professional lives, marriage, house care, and other children. Sometimes, this role takes its toll on them, becoming an obstacle to social activities, with a negative impact on their lives.

This is even more common when the children have chronic diseases and, thus, require prolonged care, increasing the overload on the life of the caregiver^(15)^. In this regard, family-focused care stands out, as it is recognized as an element of care and involved in the participation of health care in order to provide quality an safe home care^(16)^.

Regarding care transition quality for children with chronic disease, this study found a mean of 87.9 in the application of the CTM-15, a higher result than that of Brazilian and international studies applied in adults. Other Brazilian research found results from 74.5 to 69.5^(11,17)^. Nonetheless, although these studies have lower means than those found in this work, it is important to highlight that they tried to evaluate the care transition quality in adults, which may have influenced this difference in the results.

Internationally, studies from Sweden, Japan, and the United States presented means of 65.8, 66.3, and 78.5 in their evaluation of the care transition quality in adult patients^(18-20)^. In turn, concerning the assessment of care transition quality in children, a study from the United States which applied the Care Transitions Measure-3, a brief version of the CTM-15, found a mean of 83.7, suggesting that the care transition quality was higher in children health care^(10)^. Although literature has not established a cutoff point in the CTM-15 linear score, the mean presented in this study, when compared to other, indicates a good level of quality in the transition of care.

Patients with chronic diseases are the main target audience of care transition, since they need constant follow up and regular supervision^(1)^. Understanding the importance of care transition for patients with chronic diseases and conditions, as well as its influence in maintaining their quality of life, may have influence the results associated with the care transition quality in this study, which was 90.0 for patients with chronic diseases, and 84.3 for the other patients. Nonetheless, there was no difference in care transition quality between types of chronic disease, indicating that the professionals are more attentive when there is a chronic disease, regardless of the specific diagnosis of the child.

Some strategies adopted by nurses for the care transition include: discharge planning, articulation with other services, social rehabilitation aid, health education, and post-discharge follow up. As a result, the contributions of this profession to the care transition are germane, since promoting the continuity of care at home leads to better health outcomes, reduces readmissions, and improves the quality of life of the patient and their family^(1)^.

Considering the need for constant follow up in health services, the patient with chronic diseases requires more actions/strategies to guarantee the continuity of care, when compared to others without these conditions^(17)^. Thus, when the patient with a chronic condition or disease is discharged, they and their family require qualified transition from the hospital to their home, accompanied by clear guidance. When recommended, Primary Services must follow up, to prevent crises^(1)^.

Regarding the CTM-15 items, the results of this study showed the difference between patients with chronic diseases and those who did not have them in items 3 (Preferences considered when deciding where health needs are met), 12 (Had a written list of appointments or exams for the next weeks), 14 (Understand how to take medications, including quantity and times), and 15 (Understand medications’ side effects).

Some chronic disease may have stabilized signs and symptoms. To do so, understanding the care and follow up that is necessary is essential. This can reflect on the stability of the disease or on the emergence of frequent crises. In the case of care for children with chronic diseases, self-care recommendations are given to the caregivers. Therefore, they must be able to recognize the symptoms of the patient, manage consultations, exams, feeding, and medication, including schedules and adverse effects^(15)^.

Regarding the items of the instrument, items 9, “Understand health care responsibilities” and 14 “Understand how to take medications” (including quantity and times) had the highest scores. These items also were among the highest means found in other Brazilian studies^(11,17)^.

The importance of self-care and self-management of patients with chronic disease is related with giving them power over their own health, and autonomy in some aspects of their lives. The identification of signs and symptoms of crises, improved communication with health personnel, and reduced hospitalizations are some of the positive aspects of the self-monitoring of chronic disease^(21)^.

The item with the lowest mean was 15 (Understand medications’ side effects), with 67.8 in a scale from 0 to 100. This result is also in accordance with literature^(11,17)^. Care transition, when it comes to knowledge about medication and its use, can, opposed to what it may seem, be considered satisfactory, since items 13 and 14 had means of 91.2 and 94.3, respectively.

Nonetheless, in the transition, patients and families receive little guidance about the adverse effects medication may present during its use, which shows that this element should be more carefully included in recommendations for the transition from hospital to home care. Recognizing the adverse effects of medication helps preventing complications that may lead to the destabilization and later readmission of the patient. Thus, the discharge plan must include, in addition to recommendations about medication use, information about which adverse effects may take place, and how to act in this situation^(11)^.

Planning discharge is a strategy to maintain the continuity of patient care after they leave hospital care, so, the patient must be sent to their home in such a way that this continuity is guaranteed. When a discharge plan is created, the professional evaluates patient and family as a whole, considering their individuality, needs, and preferences, so they can have good results after disc charge, reducing their likelihood of being readmitted into the health services. Discharge planning aims to reeducate patient and family, preventing and dealing with complications, preparing to carry out self-care, and providing information about later procedures and consultations^(22)^.

Preferably, the discharge plan should be prepared by the multiprofessional team, so all patient needs are safely attended^(22)^. Many studies highlight the contributions of the nurse in this interdisciplinary process, especially that of the navigator nurse, professional whose role is promoting safe transitions from hospital to home^(23)^.

Regarding CTM-15 factors, the highest mean in this study was found in Factor 1, related with preparing for the self-management of the chronic disease with a mean of 91.4. This factor was also one of the highest means found in other Brazilian studies^(11,17)^. Basing professional care on hospital follow up alone hinders self-management preparations, while sharing knowledge for self-care leads to collective care, targeted at the individual needs of the patient, adapted to the language and the context of life of the family, and effectively promoting their autonomy and empowering all those involved^(21)^.

Therefore, it is important to elaborate a plan of care focused on patient and family, addressing strategies and attitudes for each individual that are related with the activity of care, to follow up, supervise, recognize, and be aware of all interventions needed in each case presented by each patient^(21)^. In this study, Factor 4, regarding the plan of care, had the lowest mean. This indicates that institutions must invest on the implementation of strategies that can make it easier for professionals to carry out health care plans.

### Study limitations

Although discussions about care transition have been increasingly necessary in health systems, it was difficult to find studies that addressed the child population. This limited the possibilities of discussing the findings of this study. Further research is necessary to advance the discussion of the topic of child care transition. This is especially true in the Brazilian context, which calls attention to the originality of this study.

### Contributions to the Field of Nursing

There was a difference in care transition quality between children who were discharged from hospitals with chronic diseases and those who did not have these diseases, according with their tutors. This allows us to understand better factors that influence this process. Thus, it becomes possible to guide workers and researchers in actions to improve care transition, especially regarding children health. This study calls attention to how care transitions are needed to guarantee the quality of care of patients in their homes, in addition to reducing hospital costs associated with readmissions.

## CONCLUSIONS

This work aimed to analyze the quality of the care transition of children and adolescents being discharged from hospital to home, considering the presence of chronic disease. The quality of the care transition was found to be satisfactory when compared to other national and international studies. Our results agreed with literature in regard to which factors and items in the instrument presented the highest and lowest means. Furthermore, there was a difference in the quality of the care transition between hospitalized patients with and without chronic diseases.

In addition to the CTM-15 general score, said difference could also be found in the factors of the instrument associated with understanding the use of medication, preferences imported, and having access to lists of exams and procedures. This suggests that those with chronic diseases found that the care transition was of a higher quality.

## Supplementary Material

0034-7167-reben-76-s2-e20220347-suppl01-pt

## Data Availability

https://doi.org/10.48331/scielodata.FBR1U7
