# Supplementary material for: Quality of child and adolescent care transitions considering the presence of chronic disease
Source: Rev Bras Enferm. 2023 May 29;76(Suppl 2):e20220347. doi: 10.1590/0034-7167-2022-0347 (PMC10226414; doi:10.1590/0034-7167-2022-0347)
Supplement: 0034-7167-reben-76-s2-e20220347-suppl01-pt [file 0034-7167-reben-76-s2-e20220347-suppl01-pt.pdf]

Paciente	idade com	Sexo	Município	Número d	dias de int	Vínculo cc	DC?	Capítulo C	tempo dia
1	14	Feminino	GF	4	4	Mãe	1	Malformaç	12
2	2	Masculino	F	1	3	Mãe	2		
3	2	Masculino	n	2	8	Tia	1	Doenças d	6
4	2	Feminino	F	2	2	Mãe	2		
5	0	Masculino	F	4	14	Mãe	1	Doenças d	0
6	0	Feminino	F	1	6	Pai	2		
7	0	Feminino	F	1	7	Mãe	2		
8	2	Masculino	F	1	2	Mãe	2		
9	3	Feminino	GF	1	2	Mãe	1	Doenças d	3
10	7	Masculino	GF	2	5	Pai	1	Doenças d	4
11	8	Masculino	F	1	8	Pai	2		
12	0	Feminino	F	1	6	Mãe	2		
13	7	Feminino	GF	1	5	Mãe	1	Doenças eı	0
14	0	Feminino	GF	2	3	Mãe	2		
15	5	Feminino	F	1	3	Mãe	1	Malformaç	5
16	3	Feminino	F	3	2	Pai	1	Doenças d	1
17	7	Feminino	GF	1	6	Mãe	1	Doenças eı	0
18	3	Masculino	F	1	2	Mãe	1	Doenças d	2
19	0	Masculino	F	1	4	Mãe	2		
20	4	Feminino	GF	1	3	Mãe	2		
21	9	Feminino	F	1	12	Mãe	1	Malformaç	5
22	2	Masculino	F	1	2	Mãe	1	Doenças d	2
23	13	Masculino	GF	1	7	Mãe	2		
24	8	Feminino	F	1	2	Pai	2		
25	1	Feminino	F	2	2	Mãe	1	Doenças d	1
26	0	Masculino	F	1	4	Mãe	1	Doenças d	0
27	2	Masculino	F	1	2	Mãe	1	Doenças d	1
28	1	Masculino	F	3	2	Mãe	1	Doenças d	1
29	1	Masculino	F	1	2	Mãe	2		
30	13	Masculino	F	1	2	Pai	1	Doenças d	13
31	7	Masculino	F	1	3	Mãe	1	Doenças d	5
32	0	Masculino	F	1	4	Mãe	2		
33	5	Feminino	F	1	10	Mãe	2		
34	0	Masculino	F	1	8	Mãe	1	Doenças d	0
35	1	Masculino	F	1	4	Pai	2		
36	0	Feminino	F	2	8	Tia	1	Doenças d	0
37	5	Feminino	F	1	4	Madrinha	2		
38	1	Masculino	F	2	11	Mãe	1	Malformaçi	1
39	4	Masculino	F	1	5	Mãe	2		
40	4	Masculino	F	1	2	Mãe	1	Doenças d	0
41	1	Masculino	F	1	8	Mãe	2		
42	1	Feminino	F	1	4	Mãe	2		
43	3	Feminino	n	1	3	Mãe	1	Malformaç	3
44	11	Feminino	F	1	4	Mãe	2		
45	15	Feminino	F	1	23	Mãe	1	Transtorno	1
46	0	Feminino	F	1	10	Mãe	2		
47	0	Feminino	F	1	9	Mãe	2		
48	0	Feminino	GF	2	4	Mãe	1	Malformaç	0

49	0	Masculino	F	2	4	Mãe	2		
50	0	Masculino	F	1	4	Mãe	2		
51	5	Masculino	F	1	3	Mãe	1	Doenças do	0
52	0	Feminino	F	1	7	Irmã	2		
53	0	Masculino	F	1	7	Pai	2		
54	9	Masculino	n	1	3	Mãe	1	Malformaç	9
55	3	Masculino	GF	1	3	Mãe	2		
56	1	Masculino	GF	3	4	Mãe	1	Doenças do	0
57	1	Masculino	F	1	2	Avó	2		
58	1	Masculino	F	1	3	Mãe	2		
59	0	Masculino	GF	2	7	Mãe	1	Doenças do	0
60	1	Masculino	GF	1	8	Mãe	2		
61	3	Masculino	F	2	6	Mãe	2		
62	4	Masculino	F	1	5	Pai	1	Doenças do	3
63	1	Feminino	F	1	3	Mãe	2		
64	3	Masculino	n	1	2	Mãe	1	Malformaç	2
65	12	Masculino	n	3	20	Mãe	1	Malformaç	12
66	2	Masculino	n	1	7	Mãe	2		
67	2	Masculino	GF	1	3	Mãe	2		
68	7	Feminino	GF	1	6	Pai	2		
69	7	Feminino	n	1	4	Mãe	1	Doenças do	6
70	7	Feminino	F	1	2	Mãe	1	Doenças do	0
71	2	Masculino	F	1	8	Mãe	1	Doenças do	1
72	1	Masculino	F	1	8	Mãe	1	Malformaçõ	0
73	12	Feminino	n	1	8	Mãe	1	Malformaç	0
74	6	Feminino	F	1	8	Mãe	1	Doenças do	0
75	12	Feminino	F	2	6	Mãe	1	Doenças do	12
76	6	Masculino	GF	1	7	Mãe	1	Doenças do	4
77	7	Feminino	F	1	7	Pai	1	Doenças do	7
78	1	Masculino	F	5	4	mãe	1	Malformaçõ	1
79	1	Masculino	F	2	4	Mãe	2		
80	3	Feminino	GF	1	7	Pai	1	Doenças do	0
81	9	Masculino	GF	1	5	Mãe	1	Doenças do	0
82	2	Masculino	GF	1	4	Mãe	1	Malformaçõ	0
83	2	Masculino	GF	1	7	Mãe	2		
84	7	Masculino	F	1	4	Mãe	1	Doenças do	5
85	11	Feminino	GF	1	7	Mãe	2		
86	1	Masculino	F	2	14	Pai	2		
87	13	Masculino	n	2	7	Mãe	1	Doenças en	3
88	4	Feminino	F	1	3	Mãe	1	Doenças do	4
89	0	Masculino	GF	1	2	Mãe	2		
90	1	Masculino	n	5	16	Mãe	1	Doenças do	1
91	3	Masculino	GF	1	4	Mãe	2		
92	1	Masculino	F	2	3	Mãe	2		
93	5	Masculino	GF	2	5	Mãe	1	Doenças en	5
94	2	Masculino	F	1	2	Mãe	1	Doenças do	1
95	8	Masculino	GF	1	6	Pai	1	Doenças do	6
96	0	Feminino	n	1	4	Mãe	2		
97	3	Feminino	GF	1	5	Pai	1	Doenças do	1
98	4	Masculino	n	2	3	Mãe	1	Doenças en	0

99	4	Masculino	F	2	2	Pai	1	Doenças dc	0
100	1	Feminino	F	3	5	Tia	2		
101	0	Feminino	GF	1	3	Mãe	1	Doenças dc	0
102	0	Masculino	GF	1	6	Mãe	2		
103	14	Feminino	F	3	15	Amiga	1	Transtorno	2
104	7	Feminino	F	1	12	Pai	1	Doenças dc	6
105	1	Feminino	GF	1	7	Mãe	2		
106	1	Masculino	F	1	7	Mãe	2		
107	8	Feminino	F	1	7	Pai	2		
108	9	Feminino	GF	1	7	Tia	2		
109	1	Masculino	F	3	3	Mãe	2		
110	7	Feminino	F	1	2	Mãe	1	Doenças dc	7
111	0	Masculino	F	1	7	Mãe	2		
112	2	Masculino	F	1	7	Mãe	1	Doenças dc	1
113	3	Masculino	F	1	5	Pai	1	Doenças dc	1
114	8	Masculino	GF	1	15	Mãe	1	Doenças dc	7
115	0	Masculino	F	2	5	Mãe	1	Doenças dc	0
116	0	Masculino	GF	3	5	Mãe	1	Doenças dc	1
117	4	Feminino	F	1	6	Mãe	2		
118	0	Masculino	GF	1	4	Mãe	2		
119	2	Masculino	n	1	1	Mãe	1	Malformaç	1
120	8	Masculino	GF	1	2	Mãe	1	Doenças dc	4
121	4	Feminino	F	1	4	Mãe	1	Doenças dc	0
122	0	Masculino	F	1	7	Pai	2		
123	3	Feminino	F	2	5	Mãe	1	Malformaç	4
124	1	Masculino	F	1	5	Pai	1	Malformaç	0
125	8	Feminino	F	1	3	Mãe	1	Doenças dc	7
126	1	Masculino	GF	2	7	Mãe	1	Doenças d	0
127	4	Masculino	F	2	3	Mãe	1	Doenças dc	1
128	4	Masculino	F	1	6	Pai	1	Malformaç	4
129	4	Feminino	F	2	2	Tia	1	Doenças dc	2
130	0	Masculino	F	2	32	Mãe	2		
131	4	Feminino	F	1	3	Mãe	1	Doenças dc	0
132	9	Masculino	F	2	10	Mãe	2		
133	3	Feminino	GF	1	3	Mãe	2		
134	3	Masculino	F	1	2	Pai	1	Malformaç	3
135	5	Masculino	GF	1	33	Mãe	2		
136	3	Masculino	GF	1	7	Mãe	1	Doenças dc	0
137	4	Feminino	n	1	3	Mãe	1	Malformaç	4
138	6	Masculino	GF	1	14	Mãe	2		
139	0	Feminino	n	1	14	Mãe	1	Malformaç	0
140	0	Masculino	F	1	90	Mãe	1	Doenças d	0
141	1	Feminino	n	1	19	Mãe	1	Doenças er	1
142	0	Masculino	F	2	10	Pai	2		
143	3	Masculino	F	1	8	Mãe	2		
144	0	Masculino	GF	1	2	Mãe	2		
145	0	Masculino	n	3	53	Mãe	1	Malformaç	0
146	8	Feminino	n	1	5	Mãe	1	Malformaç	8
147	6	Masculino	n	1	3	Mãe	1	Malformaçi	6
148	11	Masculino	F	1	1	Mãe	2		

149	6	Masculino	GF	5	5	Pai	1	Neoplasias	0
150	4	Masculino	n	1	10	Mãe	1	Neoplasias	0
151	11	Masculino	n	1	60	Tia	1	Neoplasias	8
152	8	Feminino	F	1	5	Mãe	1	Neoplasias	0
153	1	Masculino	GF	1	7	Mãe	1	Neoplasias	0
154	0	Masculino	GF	1	10	Mãe	2		
155	0	Masculino	GF	1	10	Mãe	2		
156	0	Masculino	n	2	14	Mãe	1	Doenças er	0
157	5	Feminino	GF	1	10	Mãe	2		
158	10	Feminino	n	1	1	Mãe	1	Doenças d	5
159	14	Feminino	GF	1	10	Irmão	1	Doenças d	12
160	0	Masculino	F	1	10	Mãe	2		
161	1	Masculino	GF	1	13	Mãe	2		
162	13	Masculino	F	1	4	Mãe	2		
163	14	Masculino	F	1	5	Avó	1	Doenças d	14
164	3	Feminino	n	1	2	Mãe	1	Malformaç	2
165	5	Feminino	F	1	7	Mãe	2		
166	7	Feminino	n	1	10	Mãe	1	Doenças do	7
167	0	Masculino	F	2	18	Mãe	1	Malformaç	0
168	6	Masculino	F	3	5	Mãe	1	Doenças do	5
169	6	Masculino	n	3	33	Mãe	1	Malformaç	6
170	7	Masculino	n	2	2	Mãe	1	Malformaç	2
171	11	Feminino	GF	1	10	Pai	2		
172	0	Feminino	n	2	14	Mãe	1	Malformaç	0
173	7	Feminino	GF	1	11	Pai	1	Malformaç	7
174	0	Masculino	GF	1	15	Mãe	1	Doenças d	0
175	13	Feminino	GF	2	3	Mãe	1	Doenças d	12
176	3	Masculino	GF	1	4	Mãe	1	Doenças do	3
177	1	Feminino	n	2	3	Mãe	1	Malformaç	1
178	6	Feminino	GF	1	21	Mãe	2		
179	9	Feminino	n	1	7	Mãe	1	Doenças do	0
180	12	Feminino	F	1	2	Mãe	2		
181	5	Masculino	F	1	2	Mãe	1	Doenças do	5
182	4	Masculino	GF	1	1	Mãe	1	Doenças do	3
183	0	Masculino	n	1	8	Mãe	2		
184	0	Masculino	F	1	10	Mãe	2		
185	0	Masculino	F	2	4	Mãe	1	Doenças do	0
186	0	Masculino	n	1	26	Mãe	2		
187	6	Feminino	n	1	21	Mãe	1	Malformaç	6
188	5	Masculino	F	1	1	Madrasta	1	Doenças do	4
189	14	Masculino	GF	1	5	Irmão	2		
190	8	Masculino	F	2	4	Mãe	1	Transtorno	5
191	4	Masculino	GF	1	4	Mãe	1	Doenças do	0
192	1	Masculino	GF	1	7	Mãe	1	Doenças do	0
193	0	Masculino	F	2	7	Mãe	2		
194	11	Masculino	F	2	7	Pai	2		
195	14	Masculino	F	1	2	Mãe	2		
196	14	Feminino	GF	1	1	Mãe	2		
197	0	Feminino	GF	3	7	Pai	1	Malformaç	0
198	6	Masculino	n	1	12	Mãe	1	Doenças do	5

199	0	Masculino	GF	1	10	Mãe	2		
200	2	Masculino	n	1	2	Mãe	1	Malformaç	2
201	6	Feminino	F	1	2	Avó	1	Malformaç	4
202	9	Feminino	n	1	23	Avó	1	Doenças do	6
203	4	Feminino	GF	Duas veze	12	Mãe	1	Doenças do	0
204	2	Feminino	n	1	14	Mãe	1	Doenças er	2
205	11	Feminino	GF	1	3	Mãe	1	Doenças er	3
206	2	Masculino	n	1	42	Mãe	1	Neoplasias	0
207	14	Masculino	n	4	3	Mãe	1	Neoplasias	3
208	3	Masculino	F	5	2	Mãe	1	Doenças do	1
209	0	Masculino	GF	2	4	Avó	1	Malformaç	0
210	3	Feminino	GF	1	3	Mãe	2		
211	0	Feminino	GF	1	4	Mãe	2		
212	13	Masculino	GF	1	7	Mãe	2		
213	1	Feminino	n	4	5	Mãe	1	Neoplasias	0
214	13	Feminino	F	1	3	Mãe	1	Malformaç	13
215	9	Masculino	n	1	3	Pai	1	Malformaç	9
216	8	Feminino	GF	1	3	Mãe	1	Malformaç	8
217	9	Feminino	n	1	40	Pai	2		
218	2	Feminino	n	3	7	Mãe	1	Malformaç	2
219	10	Masculino	n	1	4	Avó	1	Doenças do	0
220	8	Feminino	F	2	14	Mãe	2		
221	5	Feminino	GF	1	2	Mãe	1	Doenças do	4
222	6	Masculino	GF	1	9	Pai	1	Doenças do	1
223	1	Masculino	GF	1	7	Mãe	2		
224	9	Masculino	GF	1	4	Mãe	1	Doenças do	9
225	6	Masculino	GF	Não sabe	5	Avó	1	Neoplasias	0
226	5	Feminino	n	5	34	Pai	1	Neoplasias	0
227	9	Feminino	n	2	10	Mãe	1	Neoplasias	0
228	0	Masculino	n	2	3	Mãe	1	Malformaç	0
229	9	Feminino	GF	1	2	Mãe	2		
230	12	Masculino	n	1	5	Mãe	1	Malformaç	12
231	5	Masculino	GF	1	15	Amiga	2		
232	1	Masculino	F	2	4	Pai	2		
233	6	Masculino	F	2	3	Pai	1	Malformaç	6
234	2	Masculino	n	1	10	Mãe	1	Malformaç	2
235	1	Masculino	GF	2	6	Mãe	1	Doenças do	1
236	0	Feminino	n	1	14	Mãe	2		
237	0	Feminino	n	1	77	Pai	1	Malformaçõ	0
238	0	Feminino	n	2	21	Avó	1	Doenças do	0
239	0	Feminino	F	1	11	Mãe	1	Doenças do	0
240	8	Feminino	F	1	5	Mãe	1	Doenças do	2
241	3	Masculino	F	2	4	Mãe	1	Doenças do	2
242	0	Feminino	GF	1	7	Mãe	1	Doenças do	0
243	0	Feminino	F	1	5	Pai	2		
244	0	Masculino	F	1	6	Mãe	2		
245	2	Masculino	F	1	5	Mãe	2		
246	4	Masculino	F	1	3	Mãe	2		
247	2	Feminino	F	1	3	Mãe	2		

248	14	Feminino	F	1	12	Irmã	1	Doenças m	1
249	1	Masculino	F	1	4	Mãe	2		
250	4	Feminino	n	2	3	Mãe	1	Neoplasias	0
251	0	Masculino	n	4	17	Mãe	1	Doenças do	0
252	14	Feminino	F	2	14	Amiga	1	Transtorno	4
253	1	Feminino	GF	Bem mais,	4	Mãe	1	Neoplasias	1
254	0	Feminino	GF	1	47	Avó	1	Neoplasias	0
255	3	Feminino	F	1	9	Mãe	2		
256	5	Feminino	n	1	8	Mãe	1	Doenças do	2
257	4	Masculino	n	2	3	Mãe	1	Malformaç	0
258	3	Feminino	n	2	6	Pai	1	Doenças do	0
259	8	Masculino	GF	1	5	Mãe	1	Doenças do	0
260	7	Masculino	GF	1	5	Pai	1	Doenças do	7
261	7	Masculino	GF	1	3	Mãe	2		
262	6	Masculino	n	4	5	Mãe	1	Malformaç	4
263	0	Masculino	GF	3	60	Mãe	1	Doenças do	0
264	2	Feminino	F	1	7	Mãe	1	Malformaç	2
265	0	Masculino	GF	1	10	Mãe	1	Malformaç	0
266	0	Feminino	GF	1	11	Mãe	2		
267	9	Masculino	GF	2	2	Mãe	1	Malformaç	9
268	14	Masculino	n	1	14	Mãe	2		
269	3	Feminino	GF	4	25	Mãe	1	Doenças do	2
270	3	Masculino	GF	1	11	Avó	1	Malformaç	3
271	9	Feminino	F	1	6	Mãe	1	Doenças do	8
272	13	Feminino	n	1	14	Cuidadora	2		
273	13	Feminino	F	1	7	Madrasta	1	Transtorno	5
274	1	Masculino	GF	1	6	Mãe	1	Malformaç	1
275	7	Masculino	n	5	3	Mãe	1	Doenças do	4
276	1	Feminino	GF	1	3	Mãe	1	Malformaç	1
277	2	Feminino	F	1	6	Mãe	2		
278	13	Masculino	GF	1	8	Mãe	2		
279	4	Feminino	GF	2	7	Mãe	1	Doenças do	0
280	4	Masculino	n	2	3	Mãe	1	Doenças do	4
281	1	Masculino	GF	1	21	Mãe	1	Doenças do	0
282	0	Masculino	GF	3	4	Mãe	1	Doenças do	0
283	4	Feminino	n	Não lembra	9	Pai	1	Doenças do	3
284	9	Feminino	F	3	34	Mãe	1	Doenças do	0
285	11	Feminino	F	1	3	Mãe	2		
286	7	Feminino	GF	1	6	Mãe	2		
287	1	Feminino	GF	3	11	Mãe	2		
288	0	Masculino	GF	2	3	Mãe	2		
289	1	Masculino	n	3	21	Pai	1	Malformaç	1
290	8	Feminino	n	1	5	Mãe	1	Malformaçõ	8
291	1	Feminino	F	1	6	Mãe	1	Doenças do	1
292	8	Feminino	n	1	3	Mãe	1	Malformaçõ	8
293	11	Masculino	n	1	3	Pai	1	Malformaçõ	11
294	7	Masculino	GF	1	11	Pai	2		
295	0	Masculino	GF	1	8	Mãe	1	Malformaçõ	0
296	7	Feminino	F	1	7	Avó	2		

297	10	Masculino	n	1	17	Mãe	2		
298	7	Masculino	GF	1	5	Mãe	1	Transtorno	3
299	2	Masculino	n	1	4	Mãe	1	Malformaçõ	2
300	3	Feminino	F	1	7	Mãe	1	Doenças do	3
301	1	Feminino	n	1	31	Mãe	1	Neoplasias	0
302	0	Feminino	F	2	19	Pai	1	Neoplasias	0
303	14	Feminino	GF	1	3	Mãe	1	Neoplasias	12
304	14	Feminino	n	2	12	Mãe	1	Neoplasias	0
305	4	Feminino	GF	1	22	Mãe	1	Neoplasias	4

1. Antes d	2. A equip	3. A equip	4. Quando	5. Quando	6. Quando	7. Quando	8. Quando	9. Quando	10. Quand
3	3	3	3	3	4	4	3	4	1
4	4	3	4	4	4	3	3	4	3
4	4	4	4	4	4	4	4	4	4
3	1	3	2	4	3	4	4	4	4
4	4	4	4	4	4	4	4	4	3
4	4	4	4	4	4	4	4	4	4
3	4	4	4	4	4	3	4	4	4
4	4	4	4	4	4	3	4	4	4
3	4	4	2	2	3	4	3	3	2
3	4	4	4	4	4	4	4	3	4
3	4	3	4	4	4	4	4	4	3
3	3	4	4	4	4	4	4	4	4
4	4	4	4	4	3	4	4	4	3
3	4	4	3	3	4	4	4	4	4
3	4	3	4	4	3	3	3	3	3
3	3	2	3	3	3	2	3	3	3
4	2	4	4	4	4	4	4	4	4
3	3	3	4	4	4	3	4	3	4
3	2	3	3	3	3	3	3	3	3
3	3	3	3	3	3	3	3	3	3
3	3	3	3	4	4	3	3	3	3
4	3	3	4	3	4	3	3	3	3
3	3	3	3	3	3	3	3	4	3
4	4	4	4	4	4	4	4	4	4
3	4	4	3	4	2	3	3	3	3
3	2	3	3	3	3	2	3	3	3
4	1	1	4	4	4	4	4	4	4
4	4	4	4	4	4	4	4	4	4
4	3	3	2	2	4	3	3	3	2
4	4	4	4	4	4	2	4	4	4
4	4	4	4	4	4	4	4	4	4
3	3	3	3	3	3	3	3	3	3
3	4	4	4	4	4	4	3	4	3
4	2	3	4	3	4	4	4	3	4
3	3	3	4	4	4	3	3	3	3
0	0	0	0	0	0	0	0	0	0
4	3	3	4	4	3	4	4	4	4
4	4	4	4	4	4	4	4	4	4
3	3	3	3	4	4	4	3	4	3
4	4	4	2	3	3	2	4	3	4
4	4	4	4	4	4	4	4	4	4
3	3	2	2	3	3	3	3	3	3
3	3	4	4	4	4	4	4	4	4
4	4	4	4	4	3	4	4	4	4
4	4	4	4	4	4	4	4	4	4
4	4	4	4	4	4	4	4	4	4
4	4	4	4	4	4	2	4	4	4
3	1	3	3	3	3	3	3	3	3

2	2	2	4	4	4	3	3	4	4
3	3	2	2	2	2	3	3	3	3
4	3	4	4	4	4	4	4	4	4
1	1	1	1	1	2	2	1	4	4
3	3	3	3	3	3	3	3	3	3
4	4	4	4	4	4	4	4	4	4
3	3	3	3	3	3	3	3	3	3
4	4	4	4	4	4	4	4	4	4
3	4	4	4	4	4	4	4	4	4
3	3	4	3	3	4	4	4	4	4
4	4	3	4	4	4	4	4	4	4
4	4	4	4	4	4	4	4	4	4
4	3	4	4	4	4	4	4	4	4
4	4	4	4	4	4	4	4	4	4
3	4	2	4	4	3	3	4	4	4
4	4	4	4	4	4	4	4	4	4
4	4	4	4	4	4	4	4	4	4
4	3	3	4	4	4	4	4	4	4
4	4	4	4	4	4	4	4	4	4
2	4	4	4	4	4	4	4	4	4
4	4	3	4	3	4	3	4	4	4
4	4	4	2	1	3	1	4	4	1
4	4	4	3	3	3	4	4	4	4
4	3	3	4	4	2	4	4	4	4
4	4	3	3	4	3	4	3	4	3
4	4	4	4	4	4	4	4	4	4
3	3	4	2	4	4	4	4	4	4
4	4	4	4	4	4	4	4	4	4
4	3	4	4	4	4	4	4	4	4
4	4	4	4	4	4	4	4	4	4
4	4	4	4	4	4	4	4	4	4
4	3	4	4	4	4	4	4	4	3
4	3	4	4	4	4	4	4	4	4
4	4	4	4	4	4	4	4	4	4
3	3	2	2	3	4	2	3	3	3
3	3	3	3	3	4	4	4	3	3
4	4	4	4	4	4	4	4	4	4
4	4	3	4	4	4	4	4	4	4
4	4	4	4	4	4	4	4	4	4
4	4	4	4	4	4	4	4	4	4
3	4	4	3	4	4	4	4	4	4
4	4	4	4	4	4	4	4	4	4
4	4	2	4	4	3	4	4	4	3
4	4	4	4	4	4	4	4	4	4
4	1	4	4	4	1	4	4	4	4
3	4	4	4	4	4	4	4	4	4
4	4	4	4	4	4	4	4	4	4
4	4	4	4	4	4	4	4	4	4
4	4	4	4	4	4	4	4	4	4
4	4	4	4	4	4	4	4	4	4

4	4	4	4	4	4	4	4	4	4
4	4	4	4	4	4	4	4	4	4
4	4	4	4	4	4	4	4	4	4
4	4	4	4	4	4	4	4	4	4
4	4	4	2	3	4	4	4	4	4
4	4	4	4	4	4	4	4	4	4
4	4	4	4	4	4	4	4	4	4
4	4	4	4	4	4	4	4	4	4
4	4	4	4	4	4	4	4	4	4
4	4	4	4	4	4	4	4	4	4
3	3	2	3	3	3	4	3	3	3
4	4	4	4	4	4	4	4	4	4
2	2	3	3	3	3	2	3	3	3
4	4	4	4	4	4	4	4	4	4
4	4	4	4	4	4	4	4	4	4
4	4	4	4	4	4	4	4	4	4
4	4	4	4	4	4	4	4	4	4
2	3	4	4	4	4	4	4	4	4
3	4	3	4	4	4	4	4	4	3
4	4	4	4	4	4	4	4	4	3
4	4	4	4	4	4	0	4	4	4
4	4	4	4	4	4	4	4	4	4
3	4	4	4	4	4	4	4	4	4
3	2	4	4	4	4	4	4	4	4
4	4	4	4	4	4	4	4	4	4
4	4	4	4	4	4	4	4	4	4
3	4	4	4	4	4	4	4	4	4
4	4	2	4	4	3	3	4	4	3
2	**1**	1	4	4	4	3	4	4	4
4	4	4	4	4	4	4	4	4	4
4	4	4	4	4	4	4	4	4	4
4	4	4	4	4	4	4	4	4	4
4	4	4	4	4	4	4	4	4	4
4	4	4	4	4	4	4	4	4	3
4	4	4	4	4	4	4	4	4	4
3	3	2	4	4	4	3	4	4	4
4	4	2	4	4	4	4	4	4	4
3	4	4	4	4	4	4	4	4	4
3	4	3	4	4	4	4	4	4	4
4	4	4	4	4	4	4	4	4	4
4	4	4	4	4	4	4	4	4	4
4	4	4	4	4	4	4	4	4	3
4	4	4	4	4	4	4	4	4	4
3	1	2	3	3	3	0	1	3	1
4	4	1	4	4	4	4	2	4	4
4	4	4	4	4	4	4	4	4	2
4	4	4	3	4	3	2	3	4	2
3	3	2	4	4	4	2	3	3	4
4	4	4	4	4	4	2	4	4	3
3	4	4	4	4	4	4	4	4	4

4	4	3	4	4	4	3	4	4	4
4	4	4	4	4	4	4	4	4	4
4	4	4	4	4	4	4	4	4	4
4	4	4	4	4	4	4	4	4	4
4	4	2	4	4	4	4	4	4	3
4	4	4	4	4	4	2	4	4	4
3	3	3	3	3	4	3	4	4	3
3	3	3	3	3	3	2	3	4	3
4	4	4	4	4	4	4	4	4	4
4	4	4	4	4	4	0	4	4	4
4	4	4	4	4	4	1	4	4	4
4	4	4	4	4	4	4	3	4	4
3	4	1	4	4	4	4	4	4	4
3	4	4	4	4	4	4	4	4	4
4	4	3	4	4	4	2	4	4	4
3	3	3	3	3	3	3	3	3	3
3	4	3	3	4	3	3	3	3	3
0	0	0	0	0	0	0	0	0	0
4	3	3	4	4	4	4	4	4	4
4	4	3	4	4	4	4	4	4	4
4	3	3	4	4	4	2	4	4	3
4	3	4	4	4	4	4	4	4	4
4	4	4	4	4	4	4	4	4	4
4	4	4	4	4	4	4	3	4	4
4	4	3	4	4	4	4	4	4	4
4	2	2	4	4	2	4	4	4	4
3	3	3	3	3	3	3	3	3	3
3	4	4	4	4	4	4	4	4	4
4	4	4	4	4	4	4	4	4	4
4	4	4	4	4	4	2	4	4	4
4	4	4	4	4	4	4	4	4	4
4	4	4	4	4	4	1	4	4	4
3	4	3	4	3	3	4	3	3	3
4	3	3	4	4	4	4	4	4	4
4	4	4	4	4	4	4	4	4	4
4	4	4	4	4	4	4	4	4	2
4	4	4	4	4	4	4	4	4	4
3	2	3	4	4	3	3	3	3	4
4	4	4	4	4	4	4	4	4	4
4	4	4	4	4	4	2	3	4	4
3	3	0	4	3	4	3	4	4	3
3	3	3	3	4	3	3	3	3	3
4	4	4	4	4	4	4	4	4	4
4	4	4	4	4	4	4	4	4	4
4	4	4	4	4	4	4	4	4	4
4	4	4	4	4	4	0	4	4	4
4	4	3	4	4	4	4	3	4	4
3	2	3	3	3	3	2	3	3	2
4	4	4	4	4	4	1	3	4	2
4	3	4	4	4	4	4	4	4	4

4	3	3	4	4	4	4	4	4	4
4	4	4	4	4	4	4	4	4	4
4	4	4	4	4	4	4	4	4	4
3	3	2	2	3	4	3	4	4	3
2	2	4	4	1	2	3	2	4	2
4	4	4	4	4	4	4	4	4	4
3	4	4	4	4	4	4	4	4	4
4	4	4	4	4	4	4	4	4	4
3	4	4	3	3	4	4	4	4	4
4	4	4	4	4	4	4	4	4	4
3	3	4	2	2	3	2	2	4	4
3	3	3	3	3	3	3	3	3	3
3	2	3	3	3	3	0	0	0	0
4	4	4	4	4	4	4	4	4	4
4	4	4	4	4	4	1	4	4	4
4	4	4	4	4	4	4	4	4	4
4	4	4	4	4	4	4	4	4	4
4	4	4	4	4	4	4	4	4	4
4	4	4	4	4	4	4	4	4	4
4	4	4	4	4	4	4	4	4	4
4	4	4	4	4	4	4	4	4	4
4	4	4	2	2	4	2	4	4	4
3	3	3	4	4	4	4	4	4	4
4	4	4	4	4	4	3	4	3	4
4	4	4	4	4	4	4	4	4	4
4	1	2	4	4	4	4	4	4	4
4	4	2	4	4	4	0	4	4	4
3	4	4	3	3	3	3	4	4	3
4	4	4	4	4	4	4	4	4	4
4	4	4	4	4	3	4	4	4	4
4	3	3	2	3	3	2	3	3	3
4	4	1	4	4	4	1	4	4	4
4	0	4	4	4	4	4	4	4	4
4	4	3	4	4	4	4	4	4	4
4	4	4	4	4	4	4	4	4	4
3	4	4	2	3	4	4	3	3	3
3	4	3	3	4	4	4	4	4	3
4	1	1	4	4	1	1	4	4	2
4	3	3	2	4	4	4	4	4	4
4	4	4	4	4	4	4	4	4	4
3	3	3	4	3	4	3	4	4	3
3	3	3	3	3	3	3	3	3	3
4	4	4	3	4	4	3	3	4	4
3	4	3	4	4	3	4	3	3	4
3	3	3	3	3	3	3	3	3	3
4	2	4	4	4	4	4	4	4	4
3	3	3	3	3	3	3	3	3	3
4	4	4	4	4	4	4	4	4	4
4	4	4	4	4	4	1	4	4	4

4	3	3	4	4	4	1	4	4	4
3	3	3	3	3	3	2	4	3	3
4	4	4	4	4	4	4	3	4	4
3	3	3	4	4	4	3	4	4	4
3	3	2	2	2	1	2	2	3	3
3	4	4	4	4	4	2	4	4	3
4	4	4	4	4	4	4	4	4	4
4	4	4	4	4	4	3	4	4	4
4	4	4	4	4	4	4	4	4	4
4	4	0	4	4	4	4	4	4	4
4	4	4	4	4	4	4	4	4	4
4	4	2	4	4	4	4	4	4	4
0	4	4	4	4	4	4	4	4	3
4	4	2	4	4	4	4	4	4	4
4	4	3	4	4	4	4	4	4	4
3	3	4	4	4	4	4	4	4	4
4	4	3	4	4	4	4	3	3	3
4	4	4	3	4	3	3	4	4	4
4	4	4	4	4	4	4	4	4	4
4	4	4	4	4	4	4	4	4	4
4	4	4	4	4	4	3	4	4	4
4	4	4	4	4	4	4	4	4	4
2	4	4	4	4	4	4	4	4	4
4	4	4	4	4	4	2	4	4	4
4	4	4	4	4	4	4	4	4	4
4	4	4	4	4	4	4	4	4	4
3	4	4	3	3	4	4	3	4	3
3	4	4	3	4	4	4	3	4	4
3	2	2	4	3	4	2	4	4	2
4	4	4	4	4	4	3	4	4	4
4	4	4	4	4	4	4	4	4	4
3	4	4	4	4	3	4	4	4	4
4	4	4	4	4	4	4	4	4	4
4	4	4	4	4	4	1	4	4	4
4	4	3	4	4	2	3	4	4	4
3	4	4	4	4	4	4	4	4	4
2	2	2	2	4	4	3	3	4	3
2	2	3	4	4	4	4	4	4	4
4	4	4	4	4	4	4	4	4	4
3	3	1	2	2	1	1	2	2	1
4	4	4	4	4	4	2	4	4	4
4	4	4	4	4	4	4	4	4	4
4	4	4	4	4	4	4	4	4	4
4	4	4	4	4	4	4	4	4	4
4	4	4	4	4	4	4	4	4	4
4	4	4	4	4	4	4	4	4	4
3	4	3	4	4	2	2	4	4	4
4	4	4	4	4	4	1	4	4	4
4	3	3	4	4	4	4	4	4	4

4	4	3	4	4	4	2	4	4	4
4	4	4	4	4	4	4	4	4	4
2	2	4	4	4	4	1	4	4	4
3	0	0	3	3	3	4	3	4	4
4	4	4	4	4	4	1	4	4	4
4	4	3	2	2	4	2	2	3	4
3	4	3	3	3	4	4	4	4	3
4	4	4	4	4	4	4	4	4	3
3	4	4	4	4	4	4	4	4	4

11. Quand	12. Quand	13. Quand	14. Quand	15. Quand
2	4	4	4	3
3	3	4	4	3
4	4	4	4	4
3	2	4	4	4
4	3	4	3	3
4	4	4	4	4
4	3	4	4	3
4	2	4	4	4
2	1	0	0	0
4	3	3	4	4
3	3	4	4	3
4	4	4	4	4
4	4	4	4	4
4	4	4	4	3
3	1	4	4	4
3	2	3	3	2
4	4	4	4	4
3	2	4	4	4
3	2	3	3	3
3	3	3	3	3
3	3	3	3	3
3	4	4	3	3
4	0	4	3	2
4	3	4	4	4
3	2	3	3	3
3	2	3	3	3
3	3	4	4	2
4	4	4	4	4
3	3	3	4	1
4	4	4	4	4
4	3	4	4	4
3	3	3	3	3
4	4	4	4	2
4	4	3	4	3
3	3	3	4	1
0	0	0	0	0
3	0	4	4	4
4	4	4	4	4
3	3	3	3	3
4	4	4	4	4
4	4	4	4	4
3	3	3	3	3
4	4	4	4	4
4	2	4	4	4
4	2	3	4	3
4	4	4	4	4
4	3	4	4	2
3	3	3	3	3

4	2	3	1	1
3	2	2	2	2
4	4	2	4	4
4	1	4	4	1
3	3	4	3	3
4	4	4	4	4
3	3	3	4	3
4	4	4	4	1
4	3	4	3	4
4	2	4	4	1
4	2	4	4	4
4	4	4	4	4
4	4	4	4	4
4	4	4	4	4
3	4	3	3	2
4	4	4	4	4
3	4	3	3	3
4	2	4	4	1
4	4	4	4	1
4	4	4	4	2
4	2	4	4	1
1	1	1	1	1
3	4	2	3	1
4	4	4	4	4
3	4	4	4	3
4	4	4	4	1
4	4	4	4	4
4	4	4	4	4
4	4	4	4	1
4	4	4	4	4
4	1	3	4	1
4	4	4	4	4
4	4	1	4	3
4	4	4	4	1
3	3	2	4	2
3	0	4	4	4
4	4	4	4	3
4	4	4	4	1
4	4	4	4	4
4	4	4	4	4
4	2	3	4	1
4	4	3	4	1
4	4	3	4	1
4	1	4	4	4
4	4	4	4	1
4	4	1	4	1
4	4	4	4	4
4	2	4	4	4
4	4	3	4	1
4	3	4	4	4

4	4	4	4	4
4	4	4	4	4
4	4	0	0	0
4	4	4	4	4
4	4	4	4	3
4	4	4	4	3
4	4	4	4	4
4	1	4	4	4
4	4	4	4	4
4	4	4	4	4
3	2	2	3	2
4	4	4	4	4
3	2	3	3	2
4	0	4	4	4
4	0	4	4	3
4	4	4	4	3
4	4	0	0	0
4	4	4	4	4
4	3	3	4	4
4	1	4	4	2
4	4	4	4	4
4	2	4	4	4
4	0	4	4	4
4	0	4	4	4
4	1	4	4	4
4	0	4	4	4
4	4	4	4	4
4	0	4	4	2
4	4	4	2	1
4	4	4	4	4
4	4	4	4	4
4	4	4	4	2
4	0	4	4	3
3	2	0	0	0
4	4	4	4	4
4	3	4	4	1
4	4	0	0	0
4	4	4	4	4
4	4	4	4	4
4	2	4	4	2
4	4	4	4	4
3	4	4	4	4
4	4	4	4	4
3	0	3	3	0
4	3	0	0	0
2	0	4	4	1
3	2	0	0	0
4	4	4	4	4
3	4	4	4	4
4	4	4	4	4

3	4	4	4	4
4	4	4	4	4
4	4	4	4	4
4	4	4	4	4
3	4	0	0	0
4	4	0	0	0
4	3	3	4	3
3	3	3	3	2
4	4	4	4	4
4	4	0	0	0
4	4	4	4	4
4	4	4	1	0
4	4	4	4	4
4	4	4	4	4
4	4	0	0	0
3	3	3	3	2
3	3	3	3	3
0	0	0	0	0
4	4	4	4	4
4	3	4	4	4
3	4	0	0	0
4	4	4	4	4
4	4	4	4	4
4	4	4	4	2
3	0	4	4	4
4	4	4	4	2
3	3	3	3	3
4	4	0	0	0
4	4	4	4	4
4	4	4	4	1
4	4	4	4	4
4	4	4	4	1
4	3	3	3	3
4	4	4	4	4
3	3	3	4	2
2	2	0	0	0
4	4	4	4	4
4	3	3	3	1
4	4	4	4	4
3	0	4	4	2
4	3	3	4	3
4	0	0	0	0
4	0	4	4	4
3	4	4	4	4
4	2	4	4	2
4	4	0	0	0
4	4	0	0	0
2	4	4	4	2
4	4	4	4	1
4	4	4	4	4

4	4	4	4	4
4	4	4	4	4
4	4	4	4	4
2	4	4	4	2
4	4	2	4	2
4	4	4	4	4
4	4	4	4	4
4	4	4	4	4
4	4	4	4	4
4	4	4	4	4
4	3	4	4	1
3	3	3	4	2
0	0	0	0	0
4	4	4	4	3
4	4	4	4	4
4	4	0	0	0
4	4	4	4	4
4	4	4	4	4
4	4	0	0	0
4	4	4	4	4
4	4	4	4	4
4	4	4	4	2
3	4	4	4	3
3	4	4	4	3
4	4	4	4	0
4	4	4	4	4
4	2	4	4	4
3	4	4	4	3
4	4	4	4	4
4	4	4	4	4
3	3	3	3	2
4	4	4	4	4
4	4	0	0	0
4	0	4	4	4
4	4	4	4	4
3	3	4	4	3
3	4	4	4	3
2	3	4	4	1
3	4	4	4	3
4	4	4	4	4
4	3	0	0	0
3	3	3	3	3
3	3	4	4	4
4	3	4	4	3
3	3	3	3	3
4	0	4	4	1
3	3	0	0	0
4	0	4	4	1
4	0	0	0	0

4	0	4	4	1
3	1	4	4	1
4	4	4	4	3
4	4	4	4	4
3	2	1	3	1
2	4	4	4	4
4	4	4	4	4
4	2	2	4	3
4	4	4	4	4
4	4	4	4	4
4	4	4	4	4
4	0	4	4	3
3	4	4	4	4
4	4	4	4	2
4	4	4	4	4
4	4	4	4	4
3	3	3	3	3
4	1	4	4	3
4	4	4	4	4
4	4	4	4	4
4	4	4	4	2
4	4	4	4	2
4	4	4	4	4
4	4	4	4	4
4	4	4	4	2
4	4	4	4	4
4	4	4	4	2
3	4	3	4	2
3	2	4	4	2
4	4	4	4	2
4	4	4	4	1
4	2	4	4	4
4	4	4	4	4
4	4	0	0	0
4	3	4	4	1
4	4	3	4	4
3	4	4	4	1
4	4	4	2	1
4	4	4	4	1
1	1	4	4	1
4	4	4	4	2
4	4	4	4	4
4	4	4	4	4
4	4	4	4	4
4	0	4	4	2
4	4	4	4	4
4	2	4	4	2
4	2	0	0	0
4	0	0	0	0

4	4	2	4	2
4	4	0	0	0
4	4	4	4	4
4	1	0	0	0
4	4	4	4	1
4	4	4	4	2
3	4	4	4	2
3	4	4	4	4
4	4	3	4	4
